# 13-Hydr­oxy-4,16-dimethyl-4,16-diaza­penta­cyclo­[12.3.1.0^1,5^.0^5,13^.0^7,12^]octa­deca-7(12),8,10-triene-6,18-dione

**DOI:** 10.1107/S1600536809015293

**Published:** 2009-04-30

**Authors:** J. Suresh, K. Gurunathan, R. Suresh Kumar, S. Perumal, P. L. Nilantha Lakshman

**Affiliations:** a Department of Physics, The Madura College, Madurai 625 011, India; b School of Chemistry, Madurai Kamaraj University, Madurai 625 021, India; c Department of Food Science and Technology, Faculty of Agriculture, University of Ruhuna, Mapalana, Kamburupitiya 81100, Sri Lanka

## Abstract

In the title compound, C_18_H_20_N_2_O_3_, the *N*-methyl­piperidone ring adopts a chair conformation. The pyrrolidine ring and the five-membered cyclo­pentane rings adopt envelope conformations. The five-membered ring of the ninhydrin system adopts an envelope conformation with the central C atom deviating by 0.217 (1)Å from the mean plane through the other atoms. The mol­ecular packing is characterized by inter­molecular C—H⋯O and intra­molecular C—H⋯O and O—H⋯N inter­actions.

## Related literature

For the cytotoxic and anti­cancer properties of piperidinones, see: Dimmock *et al.* (1990 ▶, 2001 ▶). Piperidinone derivatives have attracted attention due to their predicted mode of inter­action with cellular thiols, having little or no affinity for the hydr­oxy and amino groups found in nucleic acids, see: Baluja *et al.* (1964 ▶); Mutus *et al.* (1989 ▶). Ninhydrin is used to monitor deprotection in solid phase peptide synthesis (Kaiser *et al.*, 1970 ▶). For puckering parameters, see: Cremer & Pople (1975 ▶).
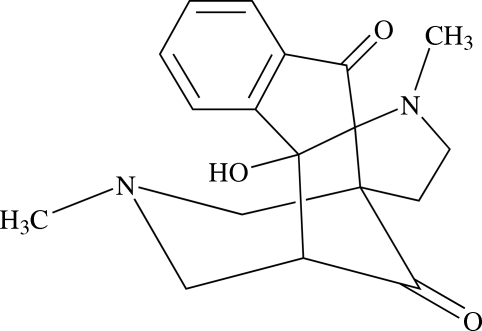


## Experimental

### 

#### Crystal data

C_18_H_20_N_2_O_3_
*M* *_r_* = 312.36
Monoclinic, 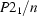

*a* = 11.0862 (5) Å
*b* = 11.6152 (5) Å
*c* = 12.5670 (6) Å
β = 102.851 (9)°
*V* = 1577.70 (12) Å^3^
*Z* = 4
Mo *K*α radiation
μ = 0.09 mm^−1^
*T* = 293 K
0.18 × 0.13 × 0.11 mm

#### Data collection

Nonius MACH-3 diffractometer
Absorption correction: ψ scan (North *et al.*, 1968 ▶) *T*_min_ = 0.984, *T*_max_ = 0.990
3226 measured reflections
2764 independent reflections
1978 reflections with *I* > 2σ(*I*)
*R*_int_ = 0.046
2 standard reflections frequency: 60 min intensity decay: none

#### Refinement

*R*[*F* ^2^ > 2σ(*F* ^2^)] = 0.039
*wR*(*F* ^2^) = 0.120
*S* = 1.04
2764 reflections
211 parameters
H-atom parameters constrained
Δρ_max_ = 0.16 e Å^−3^
Δρ_min_ = −0.18 e Å^−3^

### 

Data collection: *CAD-4 EXPRESS* (Enraf–Nonius, 1994 ▶); cell refinement: *CAD-4 EXPRESS*; data reduction: *XCAD4* (Harms & Wocadlo, 1996 ▶); program(s) used to solve structure: *SHELXS97* (Sheldrick, 2008 ▶); program(s) used to refine structure: *SHELXL97* (Sheldrick, 2008 ▶); molecular graphics: *PLATON* (Spek, 2009 ▶); software used to prepare material for publication: *SHELXL97*.

## Supplementary Material

Crystal structure: contains datablocks global, I. DOI: 10.1107/S1600536809015293/at2770sup1.cif

Structure factors: contains datablocks I. DOI: 10.1107/S1600536809015293/at2770Isup2.hkl

Additional supplementary materials:  crystallographic information; 3D view; checkCIF report

## Figures and Tables

**Table 1:** Hydrogen-bond geometry (Å, °)

*D*—H⋯*A*	*D*—H	H⋯*A*	*D*⋯*A*	*D*—H⋯*A*
O2—H2⋯N2	0.82	2.12	2.655 (2)	123
C18—H18*B*⋯O3^i^	0.96	2.39	3.288 (3)	155

Symmetry code: (i) 
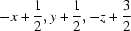
.

**Fig. 1.**
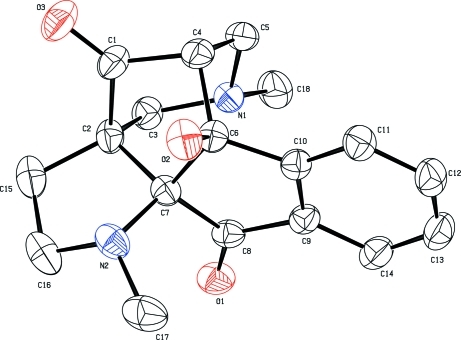
The molecular structure of the title compound, showing 30% probability displacement ellipsoids and the atom-numbering scheme.

**Fig. 2.**
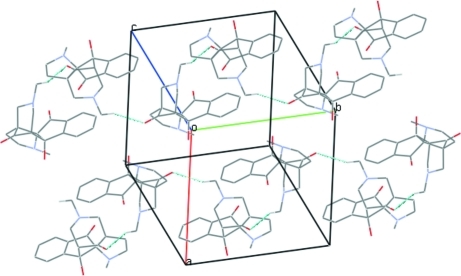
Packing diagram viewed down the c axis.

## supplementary crystallographic information

### Comment

Piperidinones belong to an important class of heterocycles which are found to possess a variety of biological activities, including cytotoxic and anticancer properties (Dimmock *et al.*, 1990, 2001). Derivatives of piperidinones have also attracted wide attention from chemists and biologists due to their predicted mode of interaction with cellular thiols, having little or no affinity for the hydroxy and amino groups found in nucleic acids (Baluja *et al.*, 1964; Mutus *et al.*, 1989). Ninhydrin is used to monitor deprotection in solid phase peptide synthesis (Kaiser *et al.*, 1970).

The molecular structure of the title compound is shown in Fig.1. The n-methyl piperidone ring adopts a chair conformation [Q=0.6668 (19) Å, θ= 13.62 (16)°, Φ= 152.0 (7)°; Cremer and Pople, 1975]. The pyrrolidine ring A(N2—C16) and the five membered cyclopentane ring B(C1—C4) adopt envelope conformations [puckering parameters Q=0.327 (2) Å, Φ=178.8 (4)° and Q=0.483 (2) Å, Φ=162.3 (2)° respectively, Cremer and Pople, 1975]. In the ninhydrin system, in the five membered ring the flap atom C7 deviate from the mean plane formed by other atoms C6/C8/C9/C10/C11/C12/C13/C14 by 0.217 (1)Å adopting an envelope conformation. The sum of the angle at the atom N2 is 338.17 (2)° is in accordance with *sp*^3^ hybridization.

Fig. 2 shows the packing viewed down the c—axis. The molecular interaction through C—H···O (Table 1) hydrogen bonds, generating a graph set motif of C_1_^1^(7) along the b—axis, stabilize the crystal structure. There are neither a marked C—H···π nor π···π interactions in the structure.

### Experimental

A mixture of 1-methyl-4-piperidinone 0.200 g (0.002 mol), ninhydrin 0.315 g (0.002 mol) and sarcosine 0.156 g (0.002 mol) in methanol (30 ml) were refluxed in a water bath for 10 h. After completion of the reaction as monitored by TLC, the excess solvent was removed under vacuum and the residue subjected to flash column chromatography using petroleum ether:ethyl acetate mixture (8:2 *v*/*v*) as eluent to obtain crystals of title compound in 8% yield along with a other product. Yield: 8%, melting point: 435–436 K.

### Refinement

The H atoms were placed in calculated positions and allowed to ride on their carrier atoms with C—H = 0.93–0.987 and Å, O—H = 0.82 Å. *U*_iso_ = 1.2*U*_eq_(C) for CH, CH_2_ groups and *U*_iso_ = 1.5*U*_eq_(C,*O*) for OH and CH_3_ groups.

### Crystal data

**Table:**

C_18_H_20_N_2_O_3_	*F*(000) = 664
*M**_r_* = 312.36	*D*_x_ = 1.315 Mg m^−^^3^
Monoclinic, *P*2_1_/*n*	Mo *K*α radiation, λ = 0.71073 Å
Hall symbol: -P 2yn	Cell parameters from 25 reflections
*a* = 11.0862 (5) Å	θ = 2–25°
*b* = 11.6152 (5) Å	µ = 0.09 mm^−^^1^
*c* = 12.5670 (6) Å	*T* = 293 K
β = 102.851 (9)°	Needle, colourless
*V* = 1577.70 (12) Å^3^	0.18 × 0.13 × 0.11 mm
*Z* = 4	

### Data collection

**Table:**

Nonius MACH-3 diffractometer	1978 reflections with *I* > 2σ(*I*)
Radiation source: fine-focus sealed tube	*R*_int_ = 0.046
graphite	θ_max_ = 25.0°, θ_min_ = 2.2°
ω–2θ scans	*h* = 0→13
Absorption correction: ψ scan (North *et al.*, 1968)	*k* = −1→13
*T*_min_ = 0.984, *T*_max_ = 0.990	*l* = −14→14
3226 measured reflections	2 standard reflections every 60 min
2764 independent reflections	intensity decay: none

### Refinement

**Table:**

Refinement on *F*^2^	Primary atom site location: structure-invariant direct methods
Least-squares matrix: full	Secondary atom site location: difference Fourier map
*R*[*F*^2^ > 2σ(*F*^2^)] = 0.039	Hydrogen site location: inferred from neighbouring sites
*wR*(*F*^2^) = 0.120	H-atom parameters constrained
*S* = 1.04	*w* = 1/[σ^2^(*F*_o_^2^) + (0.0616*P*)^2^ + 0.2971*P*] where *P* = (*F*_o_^2^ + 2*F*_c_^2^)/3
2764 reflections	(Δ/σ)_max_ < 0.001
211 parameters	Δρ_max_ = 0.16 e Å^−^^3^
0 restraints	Δρ_min_ = −0.18 e Å^−^^3^

### Special details

**Table:**

Geometry. All e.s.d.'s (except the e.s.d. in the dihedral angle between two l.s. planes) are estimated using the full covariance matrix. The cell e.s.d.'s are taken into account individually in the estimation of e.s.d.'s in distances, angles and torsion angles; correlations between e.s.d.'s in cell parameters are only used when they are defined by crystal symmetry. An approximate (isotropic) treatment of cell e.s.d.'s is used for estimating e.s.d.'s involving l.s. planes.
Refinement. Refinement of *F*^2^ against ALL reflections. The weighted *R*-factor *wR* and goodness of fit *S* are based on *F*^2^, conventional *R*-factors *R* are based on *F*, with *F* set to zero for negative *F*^2^. The threshold expression of *F*^2^ > σ(*F*^2^) is used only for calculating *R*-factors(gt) *etc*. and is not relevant to the choice of reflections for refinement. *R*-factors based on *F*^2^ are statistically about twice as large as those based on *F*, and *R*- factors based on ALL data will be even larger.

### Fractional atomic coordinates and isotropic or equivalent isotropic displacement parameters (Å^2^)

**Table:**

	*x*	*y*	*z*	*U*_iso_*/*U*_eq_	
C1	0.37990 (16)	0.05350 (16)	0.64487 (16)	0.0558 (5)	
C2	0.25684 (15)	0.03912 (14)	0.56351 (16)	0.0496 (4)	
C3	0.15940 (16)	0.06258 (15)	0.63055 (15)	0.0517 (4)	
H3A	0.0772	0.0460	0.5874	0.062*	
H3B	0.1744	0.0143	0.6951	0.062*	
C4	0.39217 (16)	0.18110 (16)	0.66421 (15)	0.0537 (5)	
H4	0.4750	0.2026	0.7051	0.064*	
C5	0.29116 (17)	0.21625 (17)	0.72388 (14)	0.0554 (5)	
H5A	0.3067	0.1796	0.7950	0.067*	
H5B	0.2935	0.2989	0.7349	0.067*	
C6	0.36690 (14)	0.22296 (15)	0.54459 (15)	0.0471 (4)	
C7	0.26099 (14)	0.14067 (15)	0.48444 (13)	0.0461 (4)	
C8	0.14614 (15)	0.21810 (16)	0.45590 (13)	0.0471 (4)	
C9	0.18837 (16)	0.33902 (15)	0.47548 (14)	0.0475 (4)	
C10	0.31366 (16)	0.34242 (15)	0.52472 (14)	0.0472 (4)	
C11	0.37277 (19)	0.44677 (16)	0.54870 (16)	0.0595 (5)	
H11	0.4564	0.4498	0.5824	0.071*	
C12	0.3053 (2)	0.54695 (17)	0.52175 (17)	0.0680 (6)	
H12	0.3445	0.6178	0.5363	0.082*	
C13	0.1808 (2)	0.54309 (18)	0.47352 (17)	0.0688 (6)	
H13	0.1369	0.6114	0.4570	0.083*	
C14	0.12088 (19)	0.43966 (18)	0.44954 (16)	0.0602 (5)	
H14	0.0370	0.4372	0.4167	0.072*	
C15	0.24197 (18)	−0.07110 (18)	0.49582 (19)	0.0671 (6)	
H15A	0.3119	−0.1222	0.5203	0.080*	
H15B	0.1667	−0.1113	0.5006	0.080*	
C16	0.2361 (2)	−0.0302 (2)	0.3805 (2)	0.0798 (7)	
H16A	0.2811	−0.0824	0.3431	0.096*	
H16B	0.1509	−0.0260	0.3397	0.096*	
C17	0.2705 (2)	0.1496 (2)	0.28884 (17)	0.0845 (7)	
H17A	0.1830	0.1563	0.2602	0.127*	
H17B	0.3075	0.1103	0.2369	0.127*	
H17C	0.3061	0.2249	0.3025	0.127*	
C18	0.06767 (19)	0.2233 (2)	0.70672 (18)	0.0693 (6)	
H18A	−0.0089	0.2123	0.6544	0.104*	
H18B	0.0787	0.3036	0.7240	0.104*	
H18C	0.0660	0.1808	0.7719	0.104*	
N1	0.16914 (13)	0.18280 (12)	0.66132 (11)	0.0479 (4)	
N2	0.29314 (14)	0.08429 (15)	0.39070 (13)	0.0603 (4)	
O1	0.04209 (10)	0.18577 (12)	0.41422 (11)	0.0618 (4)	
O2	0.47670 (10)	0.20956 (13)	0.50608 (13)	0.0650 (4)	
H2	0.4673	0.1578	0.4606	0.098*	
O3	0.45072 (13)	−0.02185 (13)	0.68415 (14)	0.0810 (5)	

### Atomic displacement parameters (Å^2^)

**Table:**

	*U*^11^	*U*^22^	*U*^33^	*U*^12^	*U*^13^	*U*^23^
C1	0.0474 (10)	0.0498 (11)	0.0671 (12)	0.0042 (8)	0.0059 (9)	0.0104 (9)
C2	0.0446 (9)	0.0392 (9)	0.0636 (11)	−0.0012 (7)	0.0093 (8)	−0.0055 (8)
C3	0.0506 (10)	0.0463 (10)	0.0576 (11)	−0.0046 (8)	0.0110 (8)	0.0019 (8)
C4	0.0431 (9)	0.0535 (11)	0.0569 (11)	−0.0048 (8)	−0.0055 (8)	0.0016 (9)
C5	0.0653 (11)	0.0536 (11)	0.0430 (9)	−0.0058 (9)	0.0026 (8)	0.0004 (8)
C6	0.0342 (8)	0.0477 (10)	0.0585 (10)	−0.0011 (7)	0.0082 (7)	0.0006 (8)
C7	0.0390 (9)	0.0495 (10)	0.0493 (10)	−0.0009 (7)	0.0089 (7)	−0.0065 (8)
C8	0.0395 (9)	0.0594 (11)	0.0416 (9)	0.0023 (8)	0.0073 (7)	0.0001 (8)
C9	0.0505 (10)	0.0509 (10)	0.0434 (9)	0.0056 (8)	0.0150 (7)	0.0053 (8)
C10	0.0504 (10)	0.0472 (10)	0.0463 (9)	−0.0013 (8)	0.0155 (8)	0.0038 (8)
C11	0.0648 (12)	0.0516 (12)	0.0635 (12)	−0.0107 (9)	0.0171 (9)	0.0023 (9)
C12	0.0982 (17)	0.0463 (12)	0.0655 (13)	−0.0053 (11)	0.0311 (12)	0.0055 (10)
C13	0.0958 (17)	0.0522 (13)	0.0640 (13)	0.0185 (11)	0.0300 (12)	0.0143 (10)
C14	0.0639 (12)	0.0639 (13)	0.0544 (11)	0.0184 (10)	0.0168 (9)	0.0104 (9)
C15	0.0553 (11)	0.0494 (11)	0.0965 (16)	−0.0015 (9)	0.0170 (11)	−0.0161 (11)
C16	0.0767 (15)	0.0750 (16)	0.0899 (17)	−0.0090 (11)	0.0229 (12)	−0.0364 (13)
C17	0.0865 (16)	0.117 (2)	0.0548 (13)	0.0058 (14)	0.0251 (11)	−0.0107 (13)
C18	0.0730 (13)	0.0697 (14)	0.0681 (13)	0.0075 (11)	0.0222 (10)	−0.0097 (11)
N1	0.0508 (8)	0.0466 (8)	0.0461 (8)	−0.0003 (6)	0.0105 (6)	−0.0013 (6)
N2	0.0567 (9)	0.0692 (11)	0.0584 (10)	0.0002 (8)	0.0201 (7)	−0.0161 (8)
O1	0.0403 (7)	0.0767 (10)	0.0626 (8)	−0.0001 (6)	−0.0011 (6)	−0.0024 (7)
O2	0.0405 (7)	0.0683 (10)	0.0891 (11)	−0.0026 (6)	0.0207 (6)	−0.0079 (8)
O3	0.0642 (9)	0.0617 (9)	0.1068 (13)	0.0116 (7)	−0.0029 (8)	0.0202 (8)

### Geometric parameters (Å, °)

**Table:**

C1—O3	1.206 (2)	C10—C11	1.379 (3)
C1—C4	1.503 (3)	C11—C12	1.384 (3)
C1—C2	1.521 (2)	C11—H11	0.9300
C2—C15	1.526 (3)	C12—C13	1.379 (3)
C2—C3	1.535 (3)	C12—H12	0.9300
C2—C7	1.549 (3)	C13—C14	1.374 (3)
C3—N1	1.446 (2)	C13—H13	0.9300
C3—H3A	0.9700	C14—H14	0.9300
C3—H3B	0.9700	C15—C16	1.513 (3)
C4—C5	1.535 (3)	C15—H15A	0.9700
C4—C6	1.545 (3)	C15—H15B	0.9700
C4—H4	0.9800	C16—N2	1.466 (3)
C5—N1	1.458 (2)	C16—H16A	0.9700
C5—H5A	0.9700	C16—H16B	0.9700
C5—H5B	0.9700	C17—N2	1.461 (3)
C6—O2	1.415 (2)	C17—H17A	0.9600
C6—C10	1.507 (2)	C17—H17B	0.9600
C6—C7	1.571 (2)	C17—H17C	0.9600
C7—N2	1.460 (2)	C18—N1	1.448 (2)
C7—C8	1.535 (2)	C18—H18A	0.9600
C8—O1	1.2154 (19)	C18—H18B	0.9600
C8—C9	1.484 (3)	C18—H18C	0.9600
C9—C14	1.387 (3)	O2—H2	0.8200
C9—C10	1.390 (2)		
			
O3—C1—C4	128.48 (17)	C11—C10—C6	128.58 (16)
O3—C1—C2	126.90 (18)	C9—C10—C6	111.31 (14)
C4—C1—C2	104.62 (14)	C10—C11—C12	118.75 (19)
C1—C2—C15	115.87 (15)	C10—C11—H11	120.6
C1—C2—C3	104.30 (15)	C12—C11—H11	120.6
C15—C2—C3	116.94 (15)	C13—C12—C11	120.90 (19)
C1—C2—C7	101.38 (13)	C13—C12—H12	119.6
C15—C2—C7	107.23 (16)	C11—C12—H12	119.6
C3—C2—C7	109.99 (14)	C14—C13—C12	120.86 (19)
N1—C3—C2	107.36 (14)	C14—C13—H13	119.6
N1—C3—H3A	110.2	C12—C13—H13	119.6
C2—C3—H3A	110.2	C13—C14—C9	118.43 (19)
N1—C3—H3B	110.2	C13—C14—H14	120.8
C2—C3—H3B	110.2	C9—C14—H14	120.8
H3A—C3—H3B	108.5	C16—C15—C2	104.31 (17)
C1—C4—C5	106.95 (15)	C16—C15—H15A	110.9
C1—C4—C6	99.36 (15)	C2—C15—H15A	110.9
C5—C4—C6	113.42 (14)	C16—C15—H15B	110.9
C1—C4—H4	112.1	C2—C15—H15B	110.9
C5—C4—H4	112.1	H15A—C15—H15B	108.9
C6—C4—H4	112.1	N2—C16—C15	105.95 (17)
N1—C5—C4	110.85 (14)	N2—C16—H16A	110.5
N1—C5—H5A	109.5	C15—C16—H16A	110.5
C4—C5—H5A	109.5	N2—C16—H16B	110.5
N1—C5—H5B	109.5	C15—C16—H16B	110.5
C4—C5—H5B	109.5	H16A—C16—H16B	108.7
H5A—C5—H5B	108.1	N2—C17—H17A	109.5
O2—C6—C10	112.31 (14)	N2—C17—H17B	109.5
O2—C6—C4	108.33 (14)	H17A—C17—H17B	109.5
C10—C6—C4	115.51 (15)	N2—C17—H17C	109.5
O2—C6—C7	112.10 (14)	H17A—C17—H17C	109.5
C10—C6—C7	104.91 (13)	H17B—C17—H17C	109.5
C4—C6—C7	103.35 (13)	N1—C18—H18A	109.5
N2—C7—C8	114.40 (14)	N1—C18—H18B	109.5
N2—C7—C2	102.91 (14)	H18A—C18—H18B	109.5
C8—C7—C2	117.03 (14)	N1—C18—H18C	109.5
N2—C7—C6	111.76 (13)	H18A—C18—H18C	109.5
C8—C7—C6	104.37 (14)	H18B—C18—H18C	109.5
C2—C7—C6	106.28 (13)	C3—N1—C18	113.60 (15)
O1—C8—C9	126.74 (16)	C3—N1—C5	113.99 (14)
O1—C8—C7	125.32 (17)	C18—N1—C5	114.19 (15)
C9—C8—C7	107.53 (13)	C7—N2—C17	116.69 (17)
C14—C9—C10	120.94 (17)	C7—N2—C16	107.45 (16)
C14—C9—C8	128.68 (16)	C17—N2—C16	114.03 (18)
C10—C9—C8	110.36 (14)	C6—O2—H2	109.5
C11—C10—C9	120.10 (17)		
			
O3—C1—C2—C15	−22.8 (3)	C6—C7—C8—O1	−175.36 (16)
C4—C1—C2—C15	157.13 (17)	N2—C7—C8—C9	−110.88 (16)
O3—C1—C2—C3	107.2 (2)	C2—C7—C8—C9	128.67 (15)
C4—C1—C2—C3	−72.83 (18)	C6—C7—C8—C9	11.57 (17)
O3—C1—C2—C7	−138.5 (2)	O1—C8—C9—C14	−1.2 (3)
C4—C1—C2—C7	41.44 (18)	C7—C8—C9—C14	171.71 (17)
C1—C2—C3—N1	66.97 (17)	O1—C8—C9—C10	−179.96 (17)
C15—C2—C3—N1	−163.63 (15)	C7—C8—C9—C10	−7.01 (19)
C7—C2—C3—N1	−41.05 (18)	C14—C9—C10—C11	0.1 (3)
O3—C1—C4—C5	−112.9 (2)	C8—C9—C10—C11	178.94 (16)
C2—C1—C4—C5	67.20 (18)	C14—C9—C10—C6	−179.82 (16)
O3—C1—C4—C6	129.0 (2)	C8—C9—C10—C6	−1.0 (2)
C2—C1—C4—C6	−50.92 (17)	O2—C6—C10—C11	−49.6 (2)
C1—C4—C5—N1	−56.50 (19)	C4—C6—C10—C11	75.3 (2)
C6—C4—C5—N1	52.0 (2)	C7—C6—C10—C11	−171.64 (18)
C1—C4—C6—O2	−80.09 (16)	O2—C6—C10—C9	130.29 (15)
C5—C4—C6—O2	166.74 (14)	C4—C6—C10—C9	−104.79 (17)
C1—C4—C6—C10	152.95 (14)	C7—C6—C10—C9	8.28 (18)
C5—C4—C6—C10	39.8 (2)	C9—C10—C11—C12	−0.7 (3)
C1—C4—C6—C7	38.98 (16)	C6—C10—C11—C12	179.21 (17)
C5—C4—C6—C7	−74.19 (17)	C10—C11—C12—C13	1.1 (3)
C1—C2—C7—N2	102.34 (15)	C11—C12—C13—C14	−0.9 (3)
C15—C2—C7—N2	−19.57 (17)	C12—C13—C14—C9	0.3 (3)
C3—C2—C7—N2	−147.71 (14)	C10—C9—C14—C13	0.1 (3)
C1—C2—C7—C8	−131.31 (15)	C8—C9—C14—C13	−178.51 (17)
C15—C2—C7—C8	106.79 (17)	C1—C2—C15—C16	−113.14 (18)
C3—C2—C7—C8	−21.4 (2)	C3—C2—C15—C16	123.18 (18)
C1—C2—C7—C6	−15.26 (17)	C7—C2—C15—C16	−0.81 (19)
C15—C2—C7—C6	−137.16 (14)	C2—C15—C16—N2	21.1 (2)
C3—C2—C7—C6	94.70 (15)	C2—C3—N1—C18	168.67 (15)
O2—C6—C7—N2	−9.8 (2)	C2—C3—N1—C5	−58.18 (19)
C10—C6—C7—N2	112.33 (16)	C4—C5—N1—C3	53.1 (2)
C4—C6—C7—N2	−126.26 (15)	C4—C5—N1—C18	−174.04 (16)
O2—C6—C7—C8	−133.98 (15)	C8—C7—N2—C17	35.0 (2)
C10—C6—C7—C8	−11.83 (17)	C2—C7—N2—C17	163.01 (16)
C4—C6—C7—C8	109.58 (15)	C6—C7—N2—C17	−83.3 (2)
O2—C6—C7—C2	101.72 (16)	C8—C7—N2—C16	−94.49 (19)
C10—C6—C7—C2	−136.13 (14)	C2—C7—N2—C16	33.53 (18)
C4—C6—C7—C2	−14.72 (16)	C6—C7—N2—C16	147.18 (16)
N2—C7—C8—O1	62.2 (2)	C15—C16—N2—C7	−35.3 (2)
C2—C7—C8—O1	−58.3 (2)	C15—C16—N2—C17	−166.26 (17)

### Hydrogen-bond geometry (Å, °)

**Table:**

*D*—H···*A*	*D*—H	H···*A*	*D*···*A*	*D*—H···*A*
O2—H2···N2	0.82	2.12	2.655 (2)	123
C15—H15A···O3	0.97	2.56	2.974 (3)	106
C18—H18B···O3^i^	0.96	2.39	3.288 (3)	155

Symmetry codes: (i) −*x*+1/2, *y*+1/2, −*z*+3/2.
